# Molecular subgroup establishment and signature creation of lncRNAs associated with acetylation in lung adenocarcinoma

**DOI:** 10.18632/aging.205407

**Published:** 2024-01-17

**Authors:** Hao Chen, Yuanyong Wang, Changjian Shao, Kai Guo, Guanglin Liu, Zhaoyang Wang, Hongtao Duan, Minghong Pan, Peng Ding, Yimeng Zhang, Jing Han, Xiaolong Yan

**Affiliations:** 1 Department of Thoracic Surgery, Tangdu Hospital of Air Force Military Medical University, Xi’an 71003, China; 2 Department of Ophthalmology, Tangdu Hospital of Air Force Military Medical University, Xi’an 71003, China

**Keywords:** lung adenocarcinoma, lncRNA, histone acetylation, prognostic signature, immunity

## Abstract

Background: The significance of long non-coding RNAs (lncRNAs) as pivotal mediators of histone acetylation and their influential role in predicting the prognosis of lung adenocarcinoma (LUAD) has been increasingly recognized. However, there remains uncertainty regarding the potential utility of acetylation-related lncRNAs (ARLs) in prognosticating the overall survival (OS) of LUAD specimens.

Methods: The RNA-Seq and clinical information were downloaded from The Cancer Genome Atlas (TCGA). Through the differential analysis, weighted correlation network analysis (WGCNA), Pearson correlation test and univariate Cox regression, we found out the prognosis associated ARLs and divided LUAD specimens into two molecular subclasses. The ARLs were employed to construct a unique signature through the implementation of the Least Absolute Shrinkage and Selection Operator (LASSO) algorithm. Subsequently, the predictive performance was evaluated using ROC analysis and Kaplan-Meier survival curve analysis. Finally, ARL expression in LUAD was confirmed by quantitative real-time PCR (qRT-PCR).

Results: We triumphantly built a ARLs prognostic model with excellent predictive accuracy for LUAD. Univariate and multivariate Cox analysis illustrated that risk model served as an independent predictor for influencing the overall survival OS of LUAD. Furthermore, a nomogram exhibited strong prognostic validity. Additionally, variations were observed among subgroups in the field of immunity, biological functions, drug sensitivity and gene mutations within the field.

Conclusions: Nine ARLs were identified as promising indicators of personalized prognosis and drug selection for people suffering with LUAD.

## INTRODUCTION

Lung cancer (LC) is a highly prevalent and lethal form of cancer worldwide, with an estimated 238,340 new cases and 127,070 deaths predicted in the United States in 2023 [1]. Among the various subtypes of LC, lung adenocarcinoma (LUAD) is considered a primary hypotype [2]. In the past few years, the molecular diagnostics, targeted therapies, and immunotherapies to LUAD has made significant progress [3]. For example, methyltransferase like 7B inhibitors can reverse tyrosine kinase inhibitors resistance in LUAD patients and serve as a feasible curative target [4]. Furthermore, USP7 was reported not only could take part in p38 MAPK pathway to influence tumor growth but also regulate PD-L1 expression in tumor and its growth environment [5]. And there are currently several tumor biomarkers and molecular markers that can are currently available to forecast the OS of people suffering with LUAD [6, 7]. Despite advancements in diagnostic and therapeutic approaches, the prognosis of LUAD maintains unsatisfactory [8]. Consequently, there is a pressing need of identifying raw biomarkers of prognosis for predicting OS of LUAD.

Histone acetylation and deacetylation plays an indispensable role in influencing chromatin adjustment and gene expression [9]. In general, histone acetylation increases chromatin flexibility and facilitates open conformation, which allows transcriptional machinery easier access to DNA [10]. Conversely, the process of histone deacetylation, which involves the removal of acetyl groups from histones is associated with compacting DNA and repressing transcription through histone deacetylase activity [11]. Histone acetylation modulator proteins (HAMPs) which composed of histone acetyltransferases (writers), histone deacetylases (erasers) and proteins containing bromodomains (readers) are major protein families for regulation and identification of histone acetylation [12]. Numerous cancers have aberrant HAMPs activity which indicating that some HAMPs may become driver genes during malignant tumorigenesis [13–15]. Unfortunately, research involving all HAMPs in LUAD is limited.

The long non-coding RNA (lncRNA) is a kind of non-protein-coding RNA that contains longer than 200 bases [16]. In recent decades, extensive research has consistently demonstrated the significant involvement of long noncoding RNAs (lncRNAs) in cancer development, as well as their contribute to plentiful biological processes like cell proliferation, differentiation, inflammation, and apoptosis [17–20]. For instance, lncRNA LINC01123 which could interact with c-Myc has been implicated in LC growth and aerobic glycolysis, while this process can also be affect by MiR-199a-5p [21]. Furthermore, lncRNA PVT1 which do duty for an oncogene can activate the KAT2A acetyltransferase and stabilize HIF-1α to modulate nasopharyngeal darcinoma [22]. However, the function of lncRNAs in regulating histone acetylation during LUAD remains ambiguous. The value of acetylation-relevant lncRNAs act as a prognostic model for people suffering with LUAD has never been systematically elucidated.

In this study, we triumphantly built a ARLs prognostic model with excellent predictive capability for LUAD. The results of univariate and multivariate Cox analysis illustrated that risk model was an isolated predictor for affecting the OS of LUAD. A nomogram reproved robust prognostic validity. There were also diversities between subgroups in the field of immunity, biological functions, drug sensitivity and gene mutations. In a word, this model may function as a biomarker and therapeutic target to forecast and prolong the prognosis of people suffering with LUAD.

## MATERIALS AND METHODS

### Data collection and preprocessing

The RNA-Seq information for LUAD and matching clinical data were obtained from the TCGA database, including 501 LUAD specimens and 56 matching paraneoplastic tissue specimens. We screened 437 LUAD specimens and 51 paraneoplastic tissue specimens according to these criteria: (1) specimens containing thorough clinical information and RNA-Seq data; (2) specimens with OS ≥30 days. These data were taken using the form of raw STAR read counts, before it was transformed by log2 and standardized in R v1.38.1. The transcriptomic data of TCGA-LUAD were separated into 3065 lncRNAs and 18158 mRNAs. Subsequently, a total of 437 LUAD samples were randomly allocated into a training cohort consisting of 306 samples and a testing cohort (131 samples) at a ratio of 7:3. Additionally, TCGA data pertaining to simple nucleotide variations (SNVs) and Copy number variation (CNV) were also obtained.

### lncRNA sifting

First of all, the DESeq2 R package was conducted to designate the differentially expressed lncRNAs (DElncRNAs) between LUAD specimens and matching paraneoplastic tissues. *P* < 0.05 and |log2FC|>1 was applied as screening cutoff. We used ggplot2 R package to depict the DElncRNAs by a volcano plot and heatmap. Next, WGCNA was adopted to select the most associated modules for LUAD. MinModuleSize was 50. Then, we searched the literature and pooled HAMPs so that obtaining a total 73 genes [23] (Supplementary Table 1). The Pearson correlation was used to determine the associations between all lncRNAs and HAMPs. As a result, we got 1065 ARLs with |correlation coefficient| >0.3 and *P* < 0.01. At last, the 55 intersecting lncRNAs of the three sets were filtrated for further investigation.

### Molecular subgroup construction

At the beginning, univariate Cox regression analysis was applied to choose 11 prognostics associated ARLs based on the previously intersecting lncRNAs. Subsequently, the ConsensusClusterPlus package was exploited to divide LUAD patients into two molecular subgroups. Principal component analysis was then conducted to assess the ability of these subgroups to effectively differentiate LUAD specimens. With the survival and survminer R packages, association between subgroups and survival condition was explored. Employing the maftools package (20), we compared the tumor mutation burden (TMB) values of subgroups. In the end, CIBERSORT arithmetic was applied to evaluate tumor immune microenvironment of subgroups.

### Building and verification of the ARLs prognostic model

The training cohort was exploited to develop the ARLs prognostic model, and the entire cohort and the testing cohort were utilized to sustain this model. LASSO analysis was subsequently executed to set up the optimal prognostic risk model based on the glment R package. As a result, a 9 ARLs prognostic model was ultimately set up. Based on the formula below, a risk score was assessed:


ris kscore=∑iExpi×coefi​

where Expi denoted lncRNA expression, and coefi represents the correlation of lncRNAs with overall survival. Through the average risk score, train queues were split into low-risk and high-risk groups. Survival analysis was performed to contrast survival rates between the two groups. ROC curve was conducted and area under the curve (AUC) at various time nodes were estimated for evaluating the prognostic worth of risk model by using the survivalROC R package.

### Differentially expressed genes (DEGs) between groups at high and low risk

Next, we analyzed distinctions in gene expression between high- and low-risk subgroups in train queues by DESq2 R package. *P*-value < 0.05 and |log2FC|>0.5 were served as screening cutoff to select the DEGs.

### Functional enrichment analysis

Furthermore, the clusterProfiler R package was employed to perform GO, KEGG and Reactome enrichment analyses were employed to identify the differentially expressed genes (DEGs). A functional comment is considered remarkably gathered when the *p*-adjust worth is less than 0.05. Then the top 15 enrichment analysis results were visualized by using bar and bubble plots. To further understand how the different biological processes engaged in subgroups. We used the clusterProfiler, enrichplot, as well as GseaVis R packages to perform gene set enrichment analysis GSEA on each sample in train cohort.

### Immunogenomic landscape analyses

The interrelationship between risk score and immune checkpoint was visualized by using the ggstatsplot package. The immunotherapy responses were also explored by using Tumor Immune Dysfunction and Exclusion (TIDE) website. We applied the CIBERSORT arithmetic to determine the quantity of 22 types of tumor-infiltrating immune cells (TIICs) in LUAD. Adopting the R package ggplot2, we displayed the diversities in tumor immune microenvironment between high-risk and low-risk teams. The StromalScore, ImmuneScore and ESTIMATEScore were calculated and showed by estimate package and ggstatsplot package.

### Drug sensitivity analysis

The Genomics of Drug Sensitivity in Cancer (GDSC) website was applied to acquire the drug-sensitivity information and the oncoPredict R package was utilized to predict whether high-risk and low-risk groups have distinct drug sensitivities. The difference between groups was presented using ggplot2 package.

### Tumor somatic mutation

We examined the disparity in SNV between high- and low-risk subgroups of LUAD. In waterfall plots, the Top 20 mutation genes were shown. The amount of gene mutations in all tumor samples in train cohort were also calculated to determine the TMB. The subsequent procedure involved conducting an analysis to investigate the relationship between TMB and risk score. Survival analysis was conducted to compare survival rates between the high and low TMB groups. Finally, CNV information was exploited to estimate copy number alterations of 9 risk lncRNAs.

### Building and proving a predictive nomogram

Multivariate and univariate Cox regression analyses were applied to inspect if the ARLs prognostic signature was an isolated element when we brought other clinical characteristics into consideration in the patients with LUAD. Then a prognostic nomogram was built in R. The validity of the nomogram signature was evaluated with the calibration curve and C-index curve.

### Cell culture and quantitative real-time polymerase chain reaction (qRT-PCR)

Human LUAD cell lines (A549) and bronchial epithelial cells (HBE) were gathered from the American Type Culture Collection (ATCC, USA), and cultured in Dulbecco’s Modified Eagle Medium (DMEM; Gibco, USA). After extracting total RNA from cells by the Trizol reagent, qRT-PCR experiments were conducted to prove the model lncRNAs.

### Availability of data and materials

In the TCGA database (https://portal.gdc.cancer.gov/), you can find the data supporting the research's results. On reasonable demand, the author may provide all data utilized in this study as well as all data supporting its findings.

## RESULTS

### Identification and screening of acetylation-related differentially expressed lncRNAs

**Figure 1:**
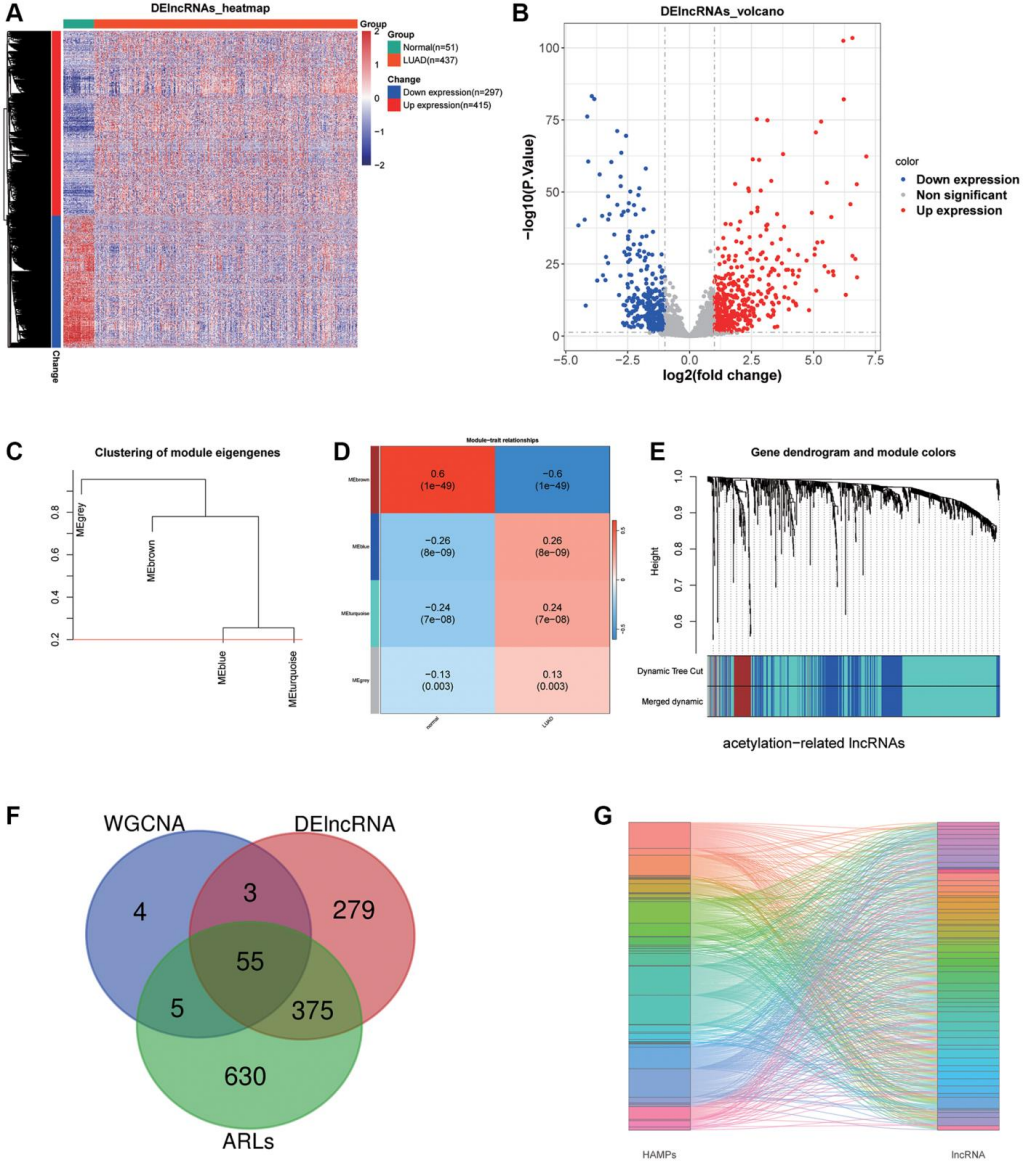
**LncRNA sifting.** (**A**, **B**) Heat map and volcano plot depicting differentially expressed lncRNAs. (**C**–**E**) WGCNA selected the most associated modules of LUAD. (**F**) A Venn graph of intersecting lncRNAs. (**G**) A Sankey diagram displays 55 acetylation-related lncRNAs.

After filtering with criteria of |log2FC|>1 and *P*-value < 0.05, 297 down expression lncRNAs and 415 up expression lncRNAs were selected between LUAD and adjoining normal tissues (Figure 1A, 1B). Then, we performed WGCNA and acquired 67 brown module lncRNAs which were the most associated with LUAD (Figure 1C–1E). Next, 1065 lncRNAs whose |correlation coefficient| >0.3 and *P* < 0.01 were prominently linked the acetylation modulator genes. Finally, 55 lncRNAs were filtrated for the next studies in total as the Venn diagram showed (Figure 1F) and their correlations with HAMPs were displayed in Figure 1G.

### ARLs molecular subgroups construction

**Figure 2:**
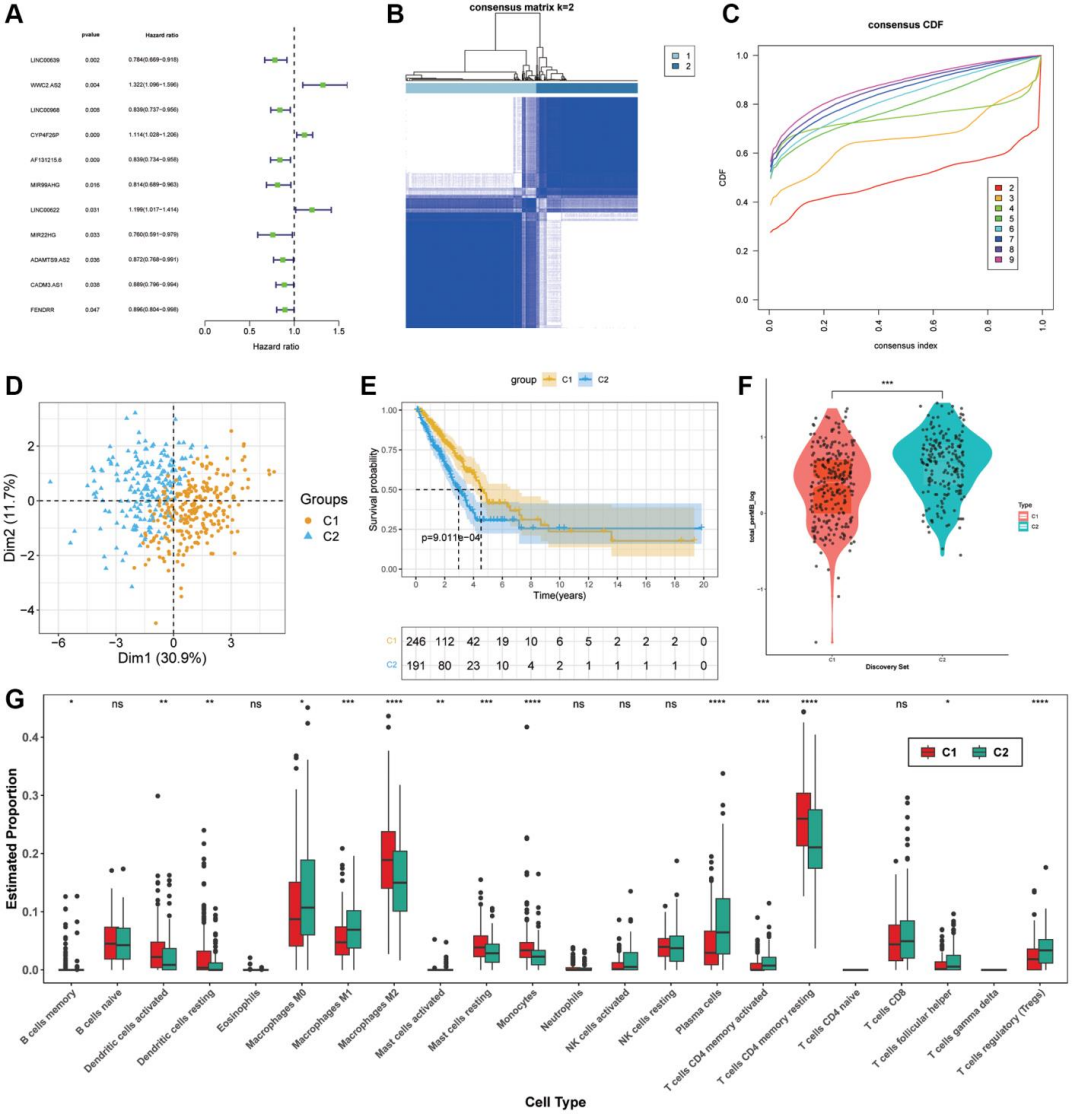
**Molecular subgroup construction.** (**A**) Prognosis related ARLs by univariate Cox regression. (**B**, **C**) Identification of two molecular subgroups. (**D**) PCA of subgroups (**E**) Kaplan-Meier survival analysis in C1, C2. (**F**) TMB levels of LUAD patients in C1 and C2. (**G**) Tumor immune microenvironment of subgroups.

For the purpose of selecting prognostic-related lncRNAs from ARLs, the univariate cox regression analysis was used and 11 ARLs (*p* < 0.05) were screened out (Figure 2A). After that, two distinct subgroups were identified by consensus cluster analysis (Figure 2B, 2C). According to PCA, these 11 ARLs had the capacity to distinguish LUAD samples precisely (Figure 2D). The KM curve demonstrated that C1 subgroup had longer OS in general (Figure 2E). In order to gain a more comprehensive understanding of variation of these two hypotypes, a comparison was made between the TMB values observed in C1 and C2. We found that mutation levels were higher in C2 (Figure 2F). Moreover, the immune cell infiltration was estimated though CIBERSORT, the outcomes revealed that C1 group had remarkably more infiltration in Dendritic cells activated, Dendritic cells resting, Macrophages M2, Mast cells resting, Monocytes as well as T cells CD4 memory resting. While C2 group had more T cells regulatory (Tregs), T cells CD4 memory activated, Plasma cells and Macrophages M0 (Figure 2G).

### Prognostic model establishment and verification of ARLs for LUAD

**Figure 3:**
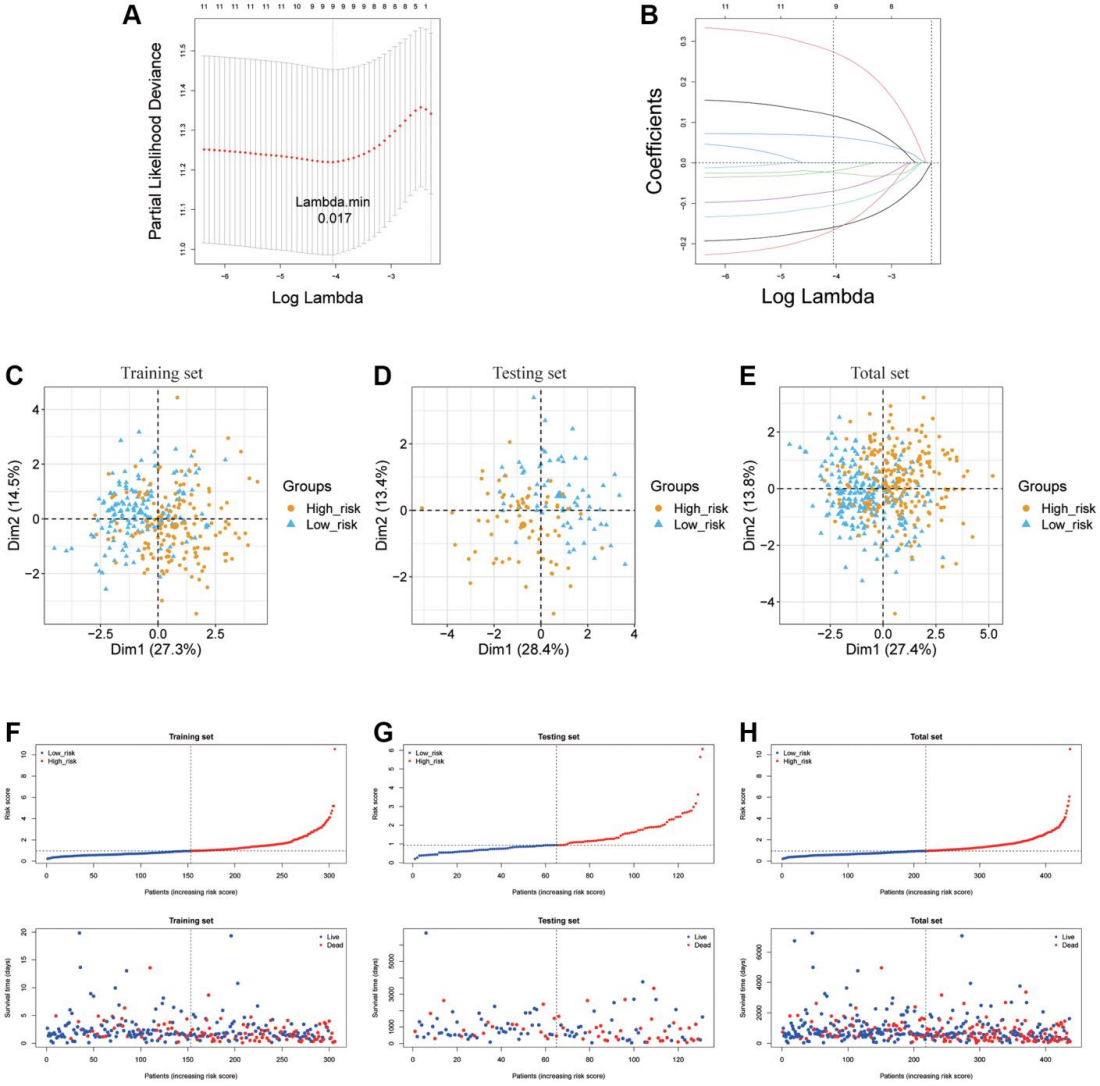
**Establishment of ARLs prognostic model.** (**A**, **B**) The LASSO of training cohort based on 11 prognoses related ARLs. (**C**–**H**) PCA and distribution of two risk groups in training testing and total cohort.

For the purpose of further examining the value of ARLs in prognosis of LUAD, LASSO was exercised to produce the optimal prognostic model (Figure 3A, 3B). As a result, nine ARLs of 11 potential survival indicators were prognostic lncRNA independently related with OS in the train cohort and were performed as a risk signature. The formula used to evaluate the risk scores of patients with LUAD was as follows:

Risk scores = 0.32965 × (WWC2-AS2) −0.18679 × (LINC00639) −0.01187 × (LINC00968) + 0.07564 × (CYP4F26P) −0.12918 × (AF131215.6) −0.09382 × (MIR99AHG) + 0.14922 × (LINC00622) −0.23000 × (MIR22HG) −0.04040 × (ADAMTS9-AS2).

**Figure 4:**
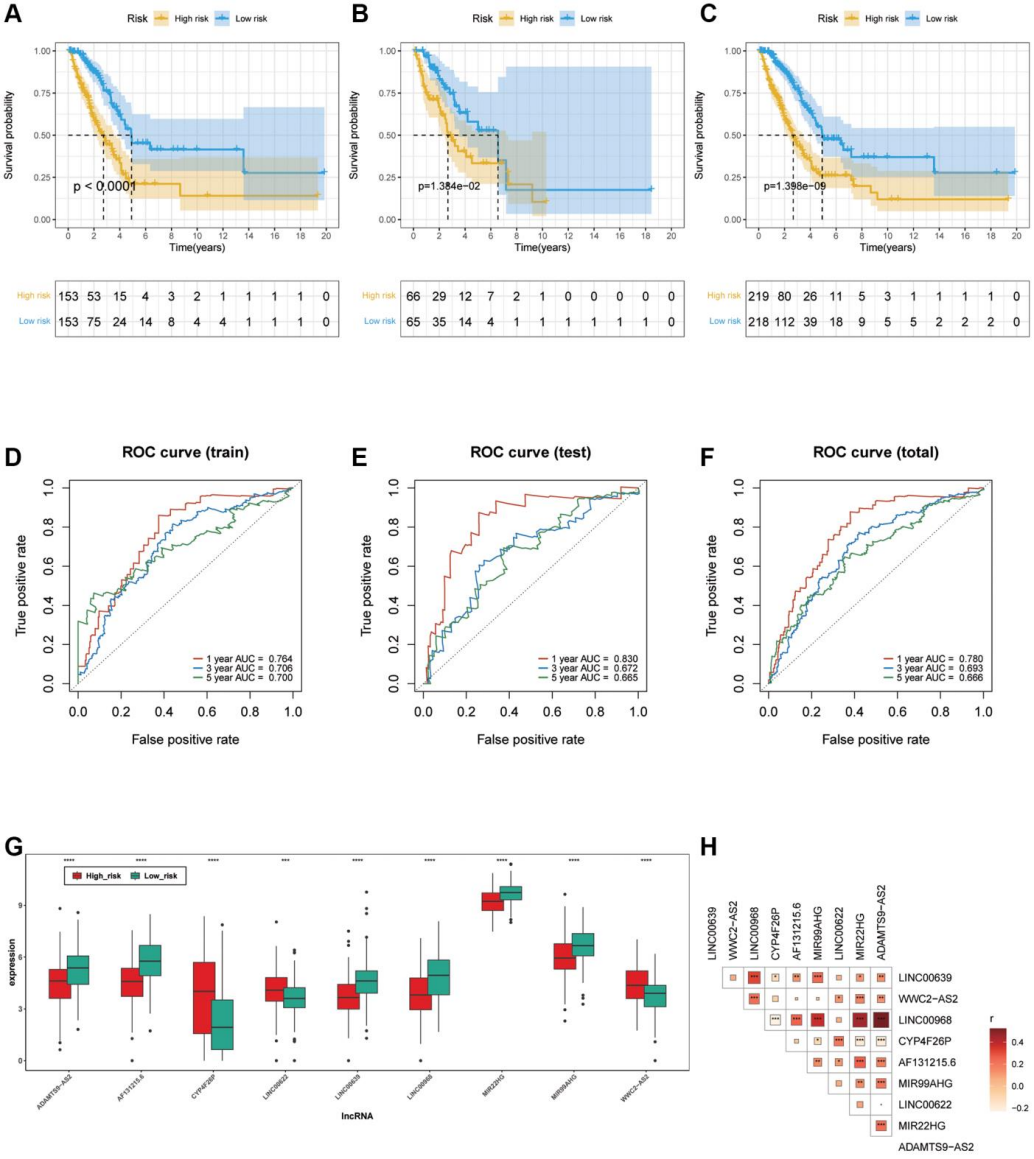
**Verification of ARLs prognostic model.** (**A**–**C**) Kaplan-Meier curve in training, testing and total cohort. (**D**–**F**) ROC curve of training, testing and total cohorts. (**G**) The expression patterns of the 9 ARLs in different subgroups. (**H**) The correlation of 9 ARLs in various groups.

The LUAD samples, in the training cohort, testing cohort and total cohort, were distributed into two risk groups using the median value of the risk scores (Figure 3C–3H). KM survival analysis manifested that the OS time of the low-risk group was remarkably longer than in the high-risk group (*p* < 0.0001 in the train cohort, *p* = 1.384e−02 in the test cohort and *p* = 1.398e−09 in the total cohort.), suggesting that the risk model of the 9 ARLs has a good prognostic worth (Figure 4A–4C). Finally, for the purpose of verifying the accuracy of the prognostic prediction of this model, the ROC curves were performed and the area under the ROC curves (AUC) was 0.764, 0.706 and 0.700 for 1-, 3-, and 5-years survival in the train set, separately (Figure 4D). As for the test cohort, the 1-, 3-, and 5-years survival AUC was 0.830, 0.672 and 0.665 (Figure 4E). Furthermore, the AUC of the total cohort was 0.780, 0.693, 0.666 for 1-, 3-, and 5-years survival (Figure 4F). In short, it indicated outstanding prediction ability of the 9-ARLs signature. Following that, we determined how the 9 ARLs are expressed in different subgroups and found that excepting CYP4F26P, other ARLs have higher rates in the low-risk group than in the high-risk group (Figure 4G). Additionally, to delve deeper into the correlation among the 9 ARLs, a Spearman analysis was performed, revealing a noteworthy co-expression association as depicted in Figure 4H.

### Differential expression and enrichment analysis

**Figure 5:**
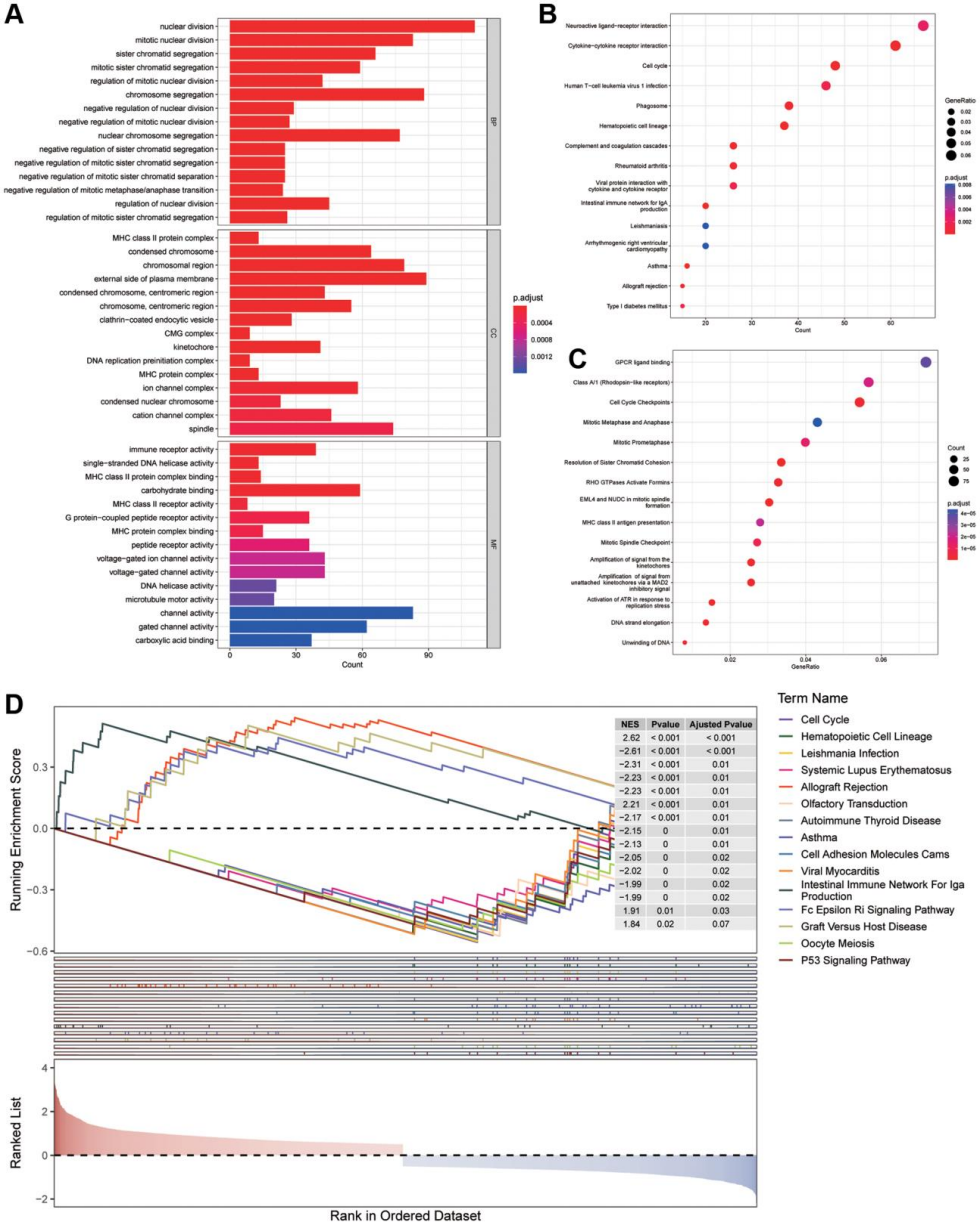
**Enrichment analysis.** (**A**) GO analysis on the biological processes (BP), cellular components (CC), and molecular functions (MF). (**B**, **C**) KEGG and Reactome enrichment pathway analysis in various subgroups. (**D**) GSEA (gene set enrichment analysis) in different subgroups.

To comprehend the differences between various risk groups, differential expression analysis was undertaken. Based on the filtering criteria of | log2FC | greater than 0.5 and *P* < 0.05, 2200 DEGs (consisting of 1085 increasing and 1115 decreasing genes) in various risk groups in the training cohort were acknowledged. Afterwards, GO, KEGG and Reactome enrichment analyses were conducted to reveal the changes in biological functions. The enrichment of MFs and CCs are mainly related to the immunity, such as MHC class II protein complex and MHC protein complex in MFs, immune receptor activity and MHC class II protein complex binding in CCs (Figure 5A). While, from the outcomes of BPs, we discovered that these DEGs had a tight association with cell cycle because they could take part in nuclear division, mitotic nuclear division and sister chromatid segregation. In addition, several KEGG and Reactome pathways revealed that the distinction in risk subgroups may have relationships with Neuroactive ligand−receptor interaction, Asthma, Amplification of signal from the kinetochores as well as Activation of ATR in response to replication stress (Figure 5B, 5C). Then, a Gene Set Enrichment Analysis (GSEA) was conducted, revealing the potential involvement of asthma, cell adhesion molecules (CAMs) and cell cycle in the observed differences between the groups (Figure 5D).

### Immunity analysis among different risk groups

**Figure 6:**
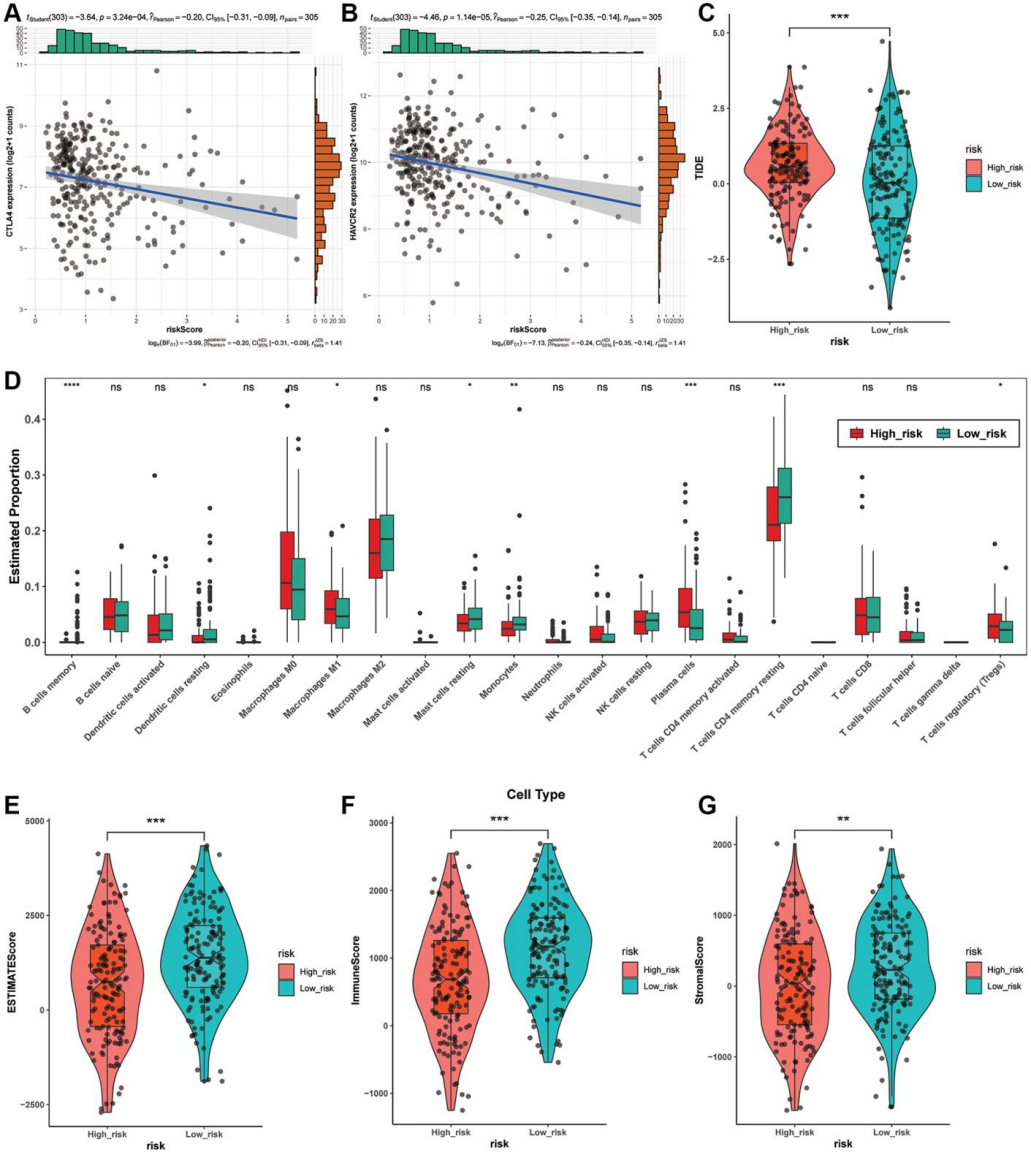
**Immunity analysis among different risk groups.** (**A**, **B**) The association between risk scores and immune checkpoints based on the Pearson test. (**C**) The TIDE levels between two risk groups. (**D**) Tumor-infiltrating immune cells in different risk groups based on CIBERSORT. (**E**–**G**) The association between StromalScore, ImmuneScore, ESTIMATEScore and risk score.

Considering the significance of immunotherapy according to checkpoints, we estimated the correlation of risk scores with immune checkpoints performing the Pearson test and discovered that risk scores were obviously related with CTLA4 (*p* = 3.24e-04), HAVCR2 (*p* = 1.14e-05) (Figure 6A, 6B). In addition, both of the two checkpoints are at higher levels in low-risk group, implying that immunotherapy aiming at them may be more effective for these individuals. Then, we estimated the effect of immunotherapy in various subgroups, and the TIDE of them had striking variation (*p* < 0.0001) (Figure 6C). Next, the correlation between risk score and StromalScore, ImmuneScore and ESTIMATEScore implied that high risk score means malignant progression of LUAD (Figure 6E–6G).

In order to investigate the potential correlation between the nine ARLs signature and tumor-infiltrating immune cells, we utilized the CIBERSORT package in R to examine the association between the risk groups and the 22 distinct types of immune cells in LUAD (Figure 6D). As the Figure 6D demonstrates, the high-risk group contained more Macrophages M1 (*p* < 0.05), Plasma cells (*p* < 0.0001) and T cells regulatory (Tregs) (*p* < 0.05) than the low-risk groups. While the low-risk group displayed more Dendritic cells resting (*p* < 0.05), than the low-risk groups. While the low-risk group displayed more Dendritic cells resting (*p* < 0.05), Mast cells resting (*p* < 0.05), T cells CD4 memory resting (*p* < 0.0001) and Monocytes (*p* < 0.01). In conclusion, these results implied that this prognostic model possibly have association with immune response by influencing immune cells.

### Significance of risk model in drug sensitivity

**Figure 7:**
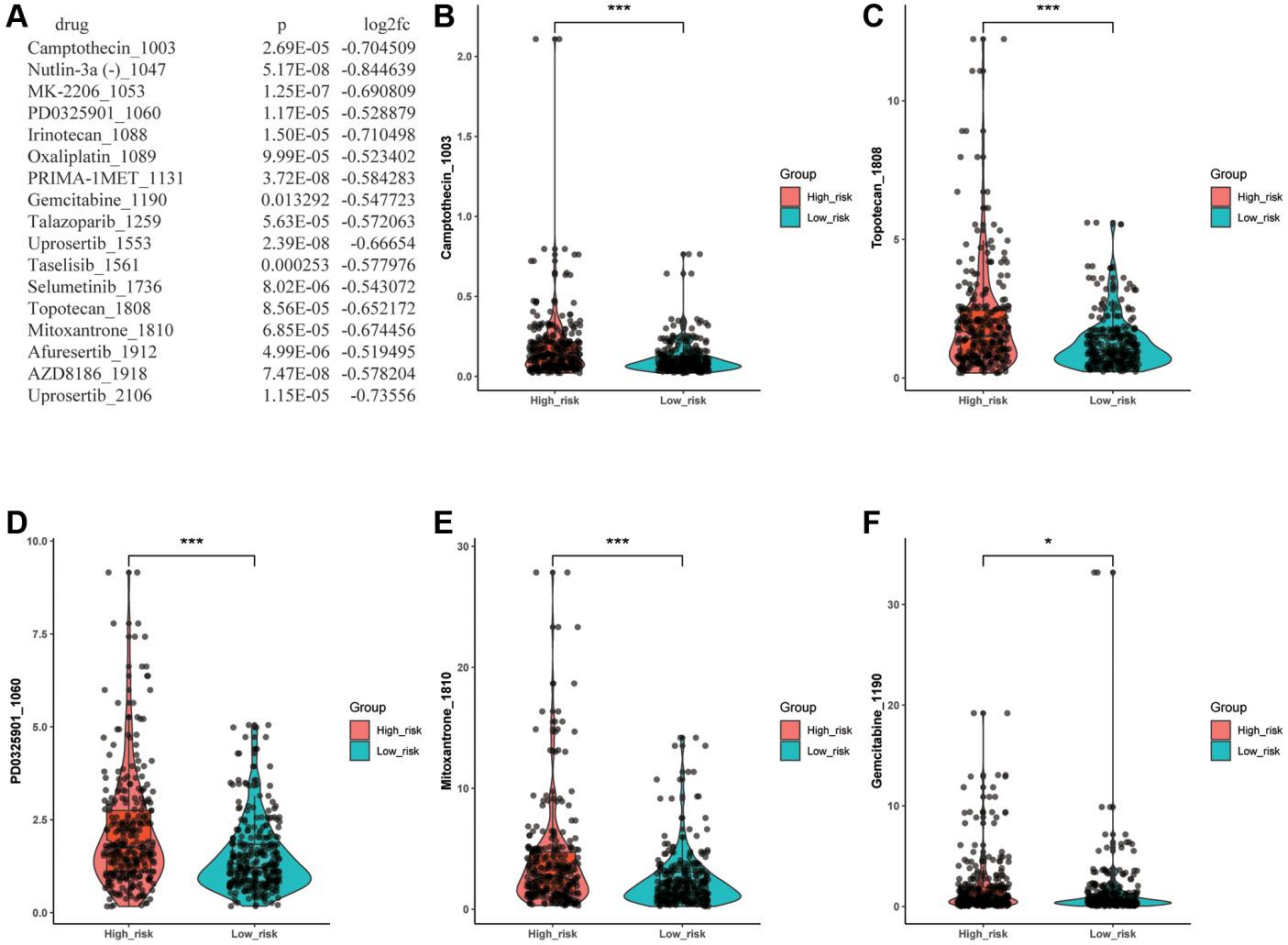
**Drug sensitivity analysis.** (**A**) Drugs with significantly different sensitivity between various groups. (**B**–**F**) The drug with IC50 <10.

To predict whether there are statistically significant different sensitivities between various risk groups to chemotherapeutic drugs, we employed the oncoPredict package and Wilcoxon test to evaluate the discrepancy. As a result, we filtrated 17 drugs which showed a significantly lower IC50 level in the low- risk group (Figure 7A). The Figure 7B–7F illustrated that 5 of 17 drugs with IC50 <10, including Camptothecin_1003, PD0325901_1060, Gemcitabine_1190, Topotecan_1808 and Mitoxantrone_1810, which meant there was a higher likelihood that these chemotherapeutic drugs would have better responses in low-risk LUAD patients. Then, according to the screening criteria for IC50 <1, Camptothecin_1003 was selected out, which indicated that the Camptothecin_1003 can possess a powerful inhibitory effect on LUAD. The results revealed that this ARLs risk signature may predict drug resistance and guide clinical treatment in LUAD patients.

### Association between the risk signature and tumor mutation status

**Figure 8:**
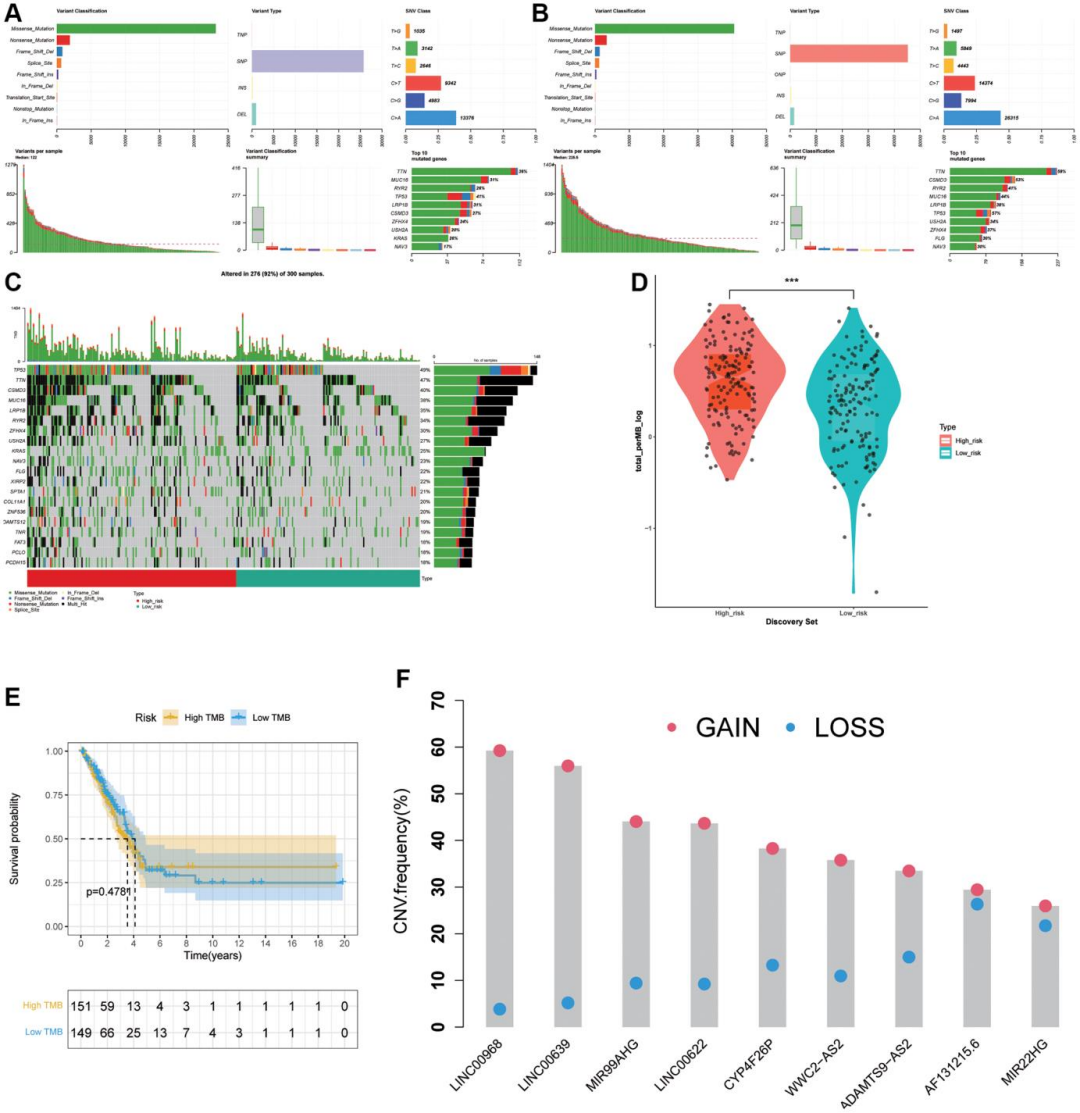
**Association between the risk signature and tumor mutation status.** (**A**) Mutation information in high-risk group. (**B**) Mutation information in low-risk group. (**C**) The top 20 mutation-rate genes in LUAD. (**D**) TMB score analysis based on risk score. (**E**) Kaplan-Meier curve in high TMB and low TMB groups. (**F**) Copy number alterations of 9 risk lncRNAs.

To analysis the panorama mutation data in LUAD, maftools package in R was utilized. Detailed mutation information in high- and low- groups was displayed in Figure 8A, 8B. Missense mutation always accounted for the highest frequency among all types of mutations in LUAD patients in various groups. Furthermore, C > A was the most general of SNV and single-nucleotide polymorphism (SNP) occurred more frequently than INS or DEL. Subsequently, we generated a waterfall plot to visualize the top 20 gene with mutation rate in LUAD (Figure 8C). In the field of TMB scores analysis, with the increasing of risk scores, TMB scores showed an obviously rise trend (Figure 8D), indicating that the ARLs risk signature is tightly related with TMB. Moreover, we further explored the association between TMB levels and prognosis but there was no apparently discrepancy (*p* = 0.4781) (Figure 8E). Next, copy number alterations of 9 risk lncRNAs were exhibited in Figure 8F.

### Independent prognostic worth of the ARLs model and establishment of a nomogram for LUAD patients

**Figure 9:**
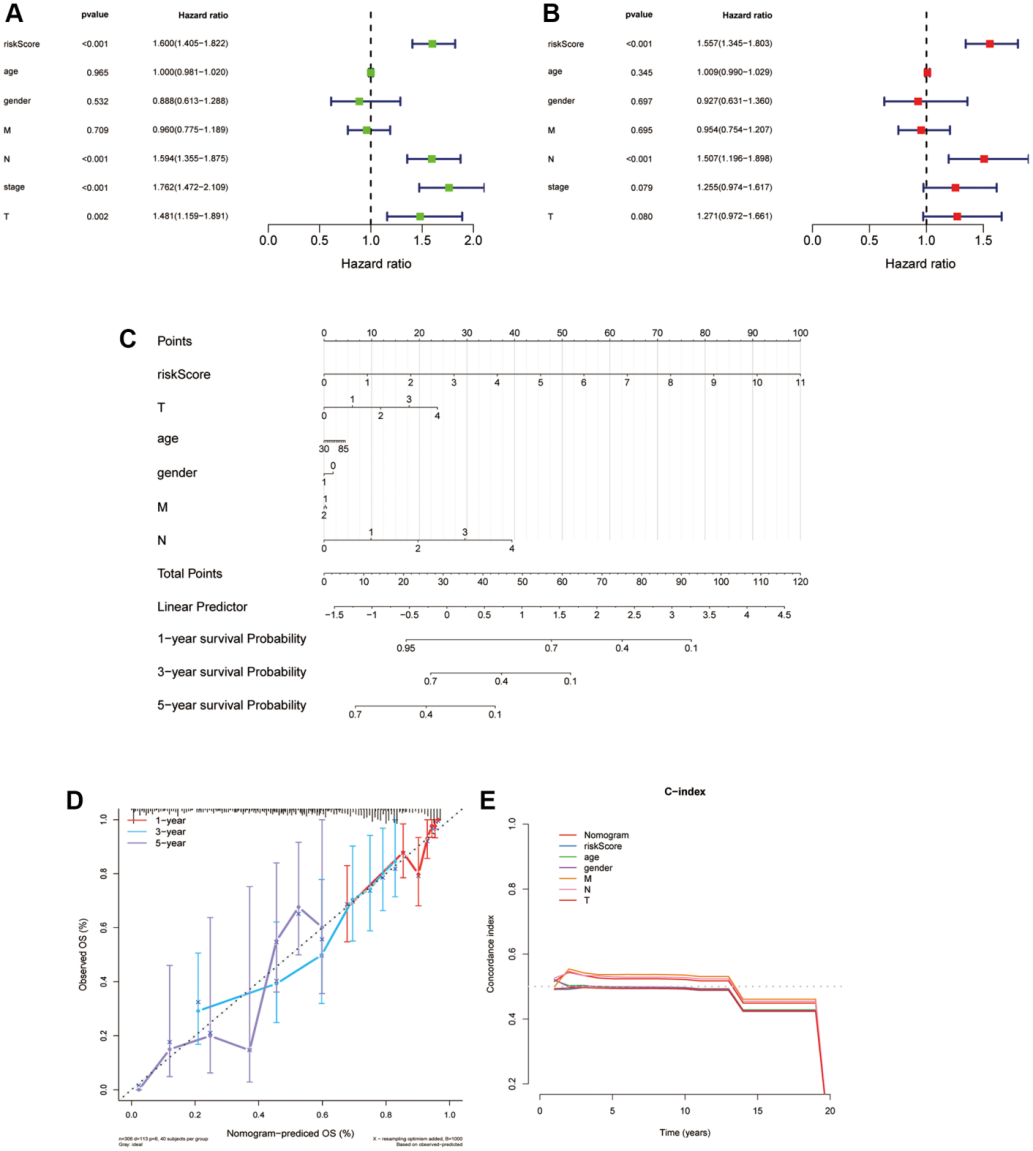
**Independent prognostic worth of the ARLs model and establishment of a nomogram for people suffering with LUAD.** (**A**, **B**) Univariate and multivariate Cox analyses of the risk score and clinical features. (**C**) Construction of a nomogram to predict OS for LUAD patients of the train cohort. (**D**, **E**) Calibration curve and c-index curve for nomogram.

We applied univariate and multivariate Cox regression analyses to find out the isolating prognostic elements in the train cohort for LUAD patients. Seven factors were included in this study, and they are risk score, age, gender, T, N, M and stage, respectively. The risk score was highly correlated with patient OS as shown in Figure 9A, 9B, which meant that it was an isolating prognostic element in patients with LUAD. In detail, according to the univariate Cox regression, risk scores are significantly associated with worse prognoses (HR = 1.600, *p* < 0.001), and the same outcome was figured out in multivariable Cox regression (HR = 1.557, *p* < 0.001). Then we built a nomogram for 1-, 3-, and 5-year OS combining the clinical characteristics and risk score to forecast the OS of LUAD patients (Figure 9C). The prognostic signature was further evaluated through calibration curves and C-index, which demonstrated the predictive capability of this particular model (Figure 9D, 9E). In conclusion, the prognostic nomogram consists of the clinical characteristics and risk score has a satisfactory manifestation in forecasting the OS of people suffering with LUAD.

### Expression verification of model lncRNAs

**Figure 10:**
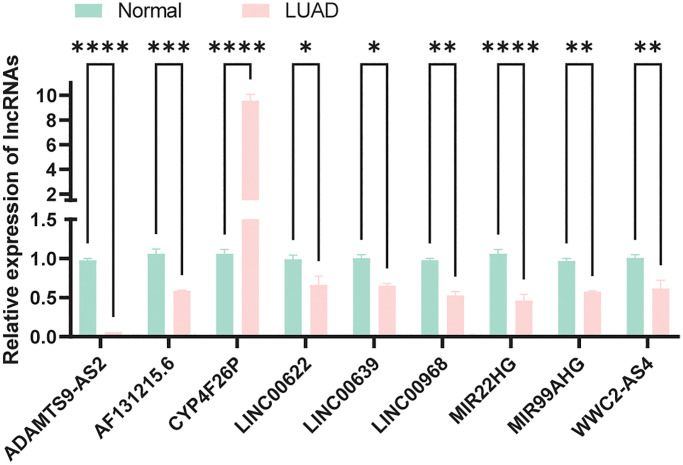
Expression verification of model lncRNAs in normal and LUAD cells.

We compared the expression of model lncRNAs between normal bronchial epithelial cells (HBE) and LUAD cells (A549) by qRT-pPCR experiment. As a result, apart from CYP4F26P, all lncRNAs were downregulated in tumor which was consistent our expectations (Figure 10) and the primer sequences were displayed in Supplementary Table 2.

## DISCUSSION

LUAD characterized by high mortality and discouraging prognosis is the primary subtype of lung cancer [24]. It is additionally a disease with heterogeneous molecular and histopathological characteristics [25]. Although it is recognized that LUAD has significantly improved in terms of surgical techniques and integrated therapies, five years survival rates are still low [26–28]. Thus, finding molecular and therapeutic targets related to LUAD prognosis in patients is a significant research area. The HAMPs play a major role in the happening and progression of numerous diseases, such as cancer [29, 30], and several drugs targeting HAMPs have been used clinically [31]. However, the investigation of lncRNAs associated with all HAMPs in the context of lung adenocarcinoma (LUAD) remains limited. It is becoming increasingly evident that lncRNAs play a crucial role in cancer prognosis [32, 33]. Nevertheless, the specific lncRNA(s) associated with HAMPs as a prognostic model in LUAD has yet to be fully elucidated.

According to the published literatures [23], 73 HAMPs were considered for this study. Based on these HAMPs, we screened out 11 prognoses related ARLs by bioinformatics analysis and distinguished two subgroups which represented appreciably distinctions in survival condition, mutation status and immune microenvironment. Then we randomly divided 437 LUAD patients into train cohort and test cohort in a 7:3 ratio. Prognostic models were constructed using the train cohort, and validation models were constructed using the test cohort and total cohort. Next, a prognostic model was establishment based on the 9 ARLs identified through univariate Cox regression and LASSO. In order to assess the accuracy of the ARLs model, we employed ROC curves and Kaplan-Meier survival analysis on the train cohort. At last, we confirmed the prognostic worth of the ARLs signature in both the test and entire sets.

Using these 9 ARLs, we developed an acetylation-associated prognostic signature. Among them, all ARLs are crucial in many ways. For instance, there is an association between WWC2-AS2 and the prognosis of multiple cancer types, such as LUAD [34], cervical cancer [35] and colon cancer [36]. Due to this, WWC2-AS2 is believed to be crucial to cancer development. Appealingly, ADAMTS9-AS2 has been widely investigated [37, 38]. Recent research showed that ADAMTS9-AS2 could restrict the deterioration of esophageal cancer and it also has the ability to function as a prognostic lncRNA in LUAD [39]. As to LINC00622, it is tightly associated with transcriptional factor androgen receptor and essential neuroblastoma progression [40]. Notably, MIR99AHG not only can influence the epithelial-mesenchymal transition in LUAD but also encourage the differentiation of bone marrow mesenchymal stem cells [41, 42]. There is evidence showing that the reduction of LINC00968 can enhance the progression of breast cancer [43]. Appealingly, MIR22HG can affect DNA damage and breast cancer growth in recent study [44, 45]. Intriguingly, LINC00639, CYP4F26P as well as AF131215.6 can fill the role of prognostic biomarker in many diseases [46–48].

For the purpose of clarifying the discrepancies in functional mechanism between high- and low-risk groups, GO, KEGG and Reactome analysis were applied. The high-risk groups exhibited involvement in pathways potentially linked to LUAD immunity, such as MHC protein complex and immune receptor activity. These findings suggest the higher risk scores is strongly correlated with immunity.

Several studies have demonstrated that immunotherapy shows clinical activity in LUAD [49, 50]. In this research, the risk scores are highly correlated with immune checkpoint expression, including HAVCR2 and CTLA4. When it comes to autoimmunity, HAVCR2 exhibits potential protection, but it is rarely expressed, while when it comes to cancer and chronic viral infections, it is often over expressed [51]. In additionally, CTLA4, the first widely used immune target in clinical practice, is mainly expressed on T cells and influences the immunocompetence of T cells [52]. The low-risk group demonstrated higher levels of these immune checkpoints, which implying that immunotherapy may be more effective in these patients. Likewise, TIDE result indicates there is significant variation between subgroups in terms of immunotherapy. Interestingly, StromalScore, ImmuneScore and ESTIMATEScore are negatively correlated with risk score. Furthermore, high-risk group have higher percentages of Macrophages M1, Plasma cells and T cells regulatory (Tregs). The number of Dendritic cells resting, Mast cells resting, T cells CD4 memory resting and Monocytes are higher in low-risk group. In summary, the observed variations in prognostic outcomes among individuals with different risk scores in LUAD can be partially explained by the relationship between risk scores and immune responses.

Predictions of drug sensitivity for LUAD patients were also made for different risk groups. People with LUAD in the high-risk group were more tolerant to Camptothecin_1003, PD0325901_1060, Gemcitabine_1190, Topotecan_1808 and Mitoxantrone_1810. While camptothecin has demonstrated a certain amount of effect in clinical tests of non-small cell lung cancer [53] and melanoma was inhibited when camptothecin combined with an HDAC inhibitor [54]. Interestingly, Gemcitabine combined with cisplatin has been proved as a practical treatment [55]. On the one hand, the 9 ARLs may be used as biomarkers to measure the effectiveness of targeted therapies. On the other hand, these findings may help in selecting clinical drugs for LUAD patients. Furthermore, there were also a positive association between TMB and risk score. TMB rates tend to be higher in groups at high risk. In some cases, this may elucidate the variation in prognosis between subgroups. Importantly, our univariate and multifactorial Cox regression analyses revealed that the risk model was an isolating prognostic element. It also improved the accuracy of forecasting the prognosis of people suffering with LUAD through the establishment of a nomogram.

There are, of course, some deficiencies to our research. At first, we explored how functional enrichment affects various risk groups, but the precise mechanisms in impacting acetylation remain to be figure out. Secondly, despite confirmation in the TCGA data, we also proved the 9 ARLs’ expression in LUAD cells with qRT-PCR. It is still necessary to externally and practically validate the prediction signature developed in our research to determine its applicability to clinical situations.

## CONCLUSIONS

In general, we shaped a robust predictive signature for lncRNAs associated with acetylation. It may function as a biomarker and therapeutic target to forecast and prolong the prognosis of people suffering with LUAD. Additionally, the model helps scientists better comprehend the relationship between acetylation and tumorigenesis. Moreover, future immunotherapies for antitumor may be explored because of this study.

## Supplementary Materials

Supplementary Tables
