# Supplementary material for: Molecular subgroup establishment and signature creation of lncRNAs associated with acetylation in lung adenocarcinoma
Source: Aging (Albany NY). 2024 Jan 17;16(2):1276–97. doi: 10.18632/aging.205407 (PMC10866443; doi:10.18632/aging.205407)
Supplement: Supplementary Tables [file aging-16-205407-s001.pdf]

SUPPLEMENTARY TABLES

Supplementary Table 1. Histone acetylation modulator proteins.

HAMPs
ASH1L
ATAD2
ATAD2B
ATAT1
BAZ1A
BAZ1B
BAZ2A
BAZ2B
BPTF
BRD1
BRD2
BRD3
BRD4
BRD7
BRD8
BRD9
BRDT
BRPF1
BRPF3
BRWD1
BRWD3
CECR2
CLOCK
CREBBP
ELP3
EP300
GTF3C4
HAT1
HDAC1
HDAC10
HDAC11
HDAC2
HDAC3
HDAC4
HDAC5
HDAC6
HDAC7
HDAC8
HDAC9
KAT2A
KAT2B

KAT5
KAT6A
KAT6B
KAT7
KAT8
KIAA2026
KMT2A
NCOA1
NCOA3
PBRM1
PHIP
SIRT1
SIRT2
SIRT3
SIRT4
SIRT5
SIRT6
SIRT7
SMARCA2
SMARCA4
SP100
SP110
SP140
SP140L
TAF1
TAF1L
TRIM24
TRIM28
TRIM33
TRIM66
ZMYND11
ZMYND8

Supplementary Table 2. Primer sequences.

Primer name	Primer sequences
ADAMTS9-AS2 F	AAGACCCACGAACGACAGC
ADAMTS9-AS2 R	CTTTCAGCCAGACATCAGGGTT
AF131215.6 F	GTGAGGATTCAAGAACCCAGGC
AF131215.6 R	GGAGGTGAGTGGAAGTGGGT
CYP4F26P F	GCTGGTTATGCTTTATGACCTGTG
CYP4F26P R	AGGGTCCATAGAGGGAGCAGAA
LINC00622 F	CCAGACATTCCCTATGCTGTTGAG
LINC00622 R	ATTTCTCACTTTCTTTAGGGCTTTTA
LINC00639 F	CTCTGATGGCGAATGTGGTCTG
LINC00639 R	CCTGAGTCCTGAAGAAGAGCACA
LINC00968 F	CATCCCATTGAGAACCAAAGAAG
LINC00968 R	CGAAAGGCTGGAAGTGTCATTAG
MIR22HG F	CAGTGATTTGCTCCCCCTCG
MIR22HG R	AGCCCATTTCTGTCACCTTCCA
MIR99AHG F	GGACAACCATAGGCAAAACTGAA
MIR99AHG R	AGTGTGCTATTTTCTGCCCCTG
WWC2-AS2 F	CGCTTTGACCGCATTTAGGG
WWC2-AS2 R	ACCAGGGCGTCTCATTCCA
GAPDH F	CTGACTTCAACAGCGACACC
GAPDH R	TGCTGTAGCCAAATTCGTTGT
